# Improving the Biocompatibility and Energy Performance of a Microbial Bioanode with Black Phosphorus and Polypyrrole Nanocomposites

**DOI:** 10.1021/acsomega.5c12114

**Published:** 2026-07-14

**Authors:** João Carlos de Souza, Ana Clara Bonizol Zani, Bruna dos Santos Gomes, Gustavo Silveira Toldo, Valeria Reginatto, Adalgisa Rodrigues de Andrade

**Affiliations:** † University of São Paulo (USP), Faculty of Philosophy, Sciences and Letters at Ribeirão Preto (FFCLRP), Department of Chemistry, Avenida Bandeirantes, 3900, Ribeirão Preto, São Paulo 14040-900, Brazil; ‡ National Institute for Identification, Quantification, Dispersion, Environmental Risks and Mitigation of Pollution by Emerging Contaminants in Marine and Coastal Environments. INCT − CEMAR, Rua Síria, 51, São Vicente, São Paulo 11330-140, Brazil

## Abstract

When it comes to enhancing the way bioelectrochemical systems perform, improving how microorganisms and electrodes interact remains challenging. Here, we have modified anodes to improve the performance of microbial fuel cells (MFCs). Specifically, we modified a graphite plate anode (GP) by using three strategies—electroactivation (EGP), electroactivation followed by black phosphorus incorporation (EGP/BP), and subsequent EGP/BP electropolymerization with polypyrrole (EGP/BP/PPy)—and evaluated EGP, EGP/BP, and EGP/BP/PPy in dual-chamber MFCs. The MFC anodic biofilm was formed from mangrove sediment in sodium acetate. Scanning electron microscopy confirmed that the modification strategies modified the GP surface and that a robust biofilm emerged after each treatment. The highly modified EGP/BP/PPy performed the best and reached a maximum voltage of 530.0 ± 35 mV, compared to 100.0 ± 18 mV and 10.8 ± 2 mV achieved with EGP/BP and EGP, respectively. EGP/BP/PPy also provided the highest maximum power density and Coulombic charge. Modifying the GP surface influenced the bacterial community composition. The genus *Alcaligenes* predominated under all the tested conditions, but other electroactive genera such as *Pseudomonas* and *Geobacter* emerged in EGP/BP/PPy. Therefore, combining BP and PPy to functionalize GP provided a stable and conductive electroactive biofilm, which enhanced bacterial adhesion, electron transfer, and energy generation. This work highlights that synergistic electrode functionalization improves electrode conductivity and microbial activity, which paves the way for advanced biohybrid electrochemical systems to be designed.

## Graphical abstract

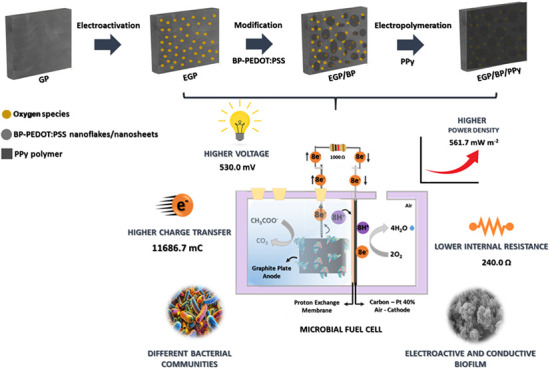

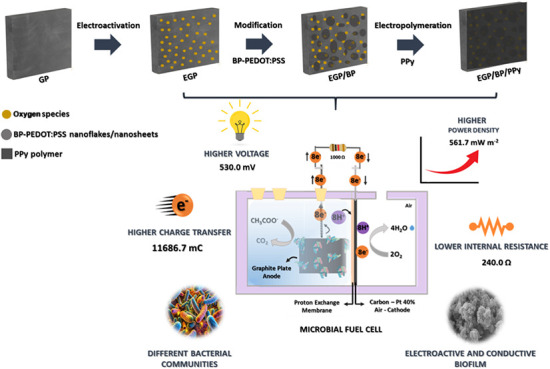

## Introduction

Functionalizing carbon surfaces is useful for improving how bioelectrochemical devices perform in terms of energy.^1–3^ By using microorganisms as biocatalysts, microbial fuel cells (MFCs) convert chemical energy originating from substrate oxidation into electrical energy. Recent investigation in this field has combined microbial catalysis with application of novel materials to the anode or cathode.^4,5^ Although MFCs perform well at the laboratory scale, controlling extracellular electron transfer (EET) represents a bottleneck for large-scale applications.^6^

Various approaches have been developed to understand the microorganism-electrode interface and to optimize EET.^6^ Such approaches include modifying the electrode surface with metallic nanoparticles or reduced graphene oxide.^1,6–8^ For a thermodynamically favorable electron transfer to occur, the electrode potential must be aligned with the microbial donor redox potential.^9^ Hence, building an efficient biohybrid system requires that different types of EET be understood, and that energy generation be improved.^6^

Direct EET deals with electrons being transferred via membrane proteins and/or conductive nanowires in physical contact with the electrode, whereas indirect EET relies on endogenously produced or externally supplied redox mediators. In addition, extracellular polymeric substances can be formed and serve as a transitional medium for mediated EET.^6,10^ Well-known electroactive bacteria such as *Shewanella* perform both mechanisms; some of these bacteria secrete flavins that act as electron shuttles, whereas others employ the metal reduction pathway to achieve direct electron transfer.^11,12^

Although electroactive microorganisms are now better understood, many bacterial genera remain poorly explored, including *Alcaligenes*, which we identified as the dominant genus in this study. Yu et al.^13^ used Fourier-transform infrared spectroscopy to investigate the electron transfer process in *Alcaligenes faecalis* biofilms. The authors observed characteristic bands attributed to cytochrome c, suggesting involvement of redox-active proteins in electrode electron transfer.

Black phosphorus (BP), a layered material that is held together by van der Waals forces, is highly conductive and mobile during charge transport, but it can be degraded under certain environmental conditions.^14,15^ To stabilize BP, it can be combined with poly­(3,4-ethylenedioxythiophene)-poly­(styrenesulfonate) (PEDOT:PSS).^16,17^ BP has conductive properties that have been enhanced by combining it with other materials. Currently, BP has been explored across a wide range of applications, including batteries, supercapacitors, sensors, transistors, photovoltaic cells, and devices for healthcare applications.^18,19^

Polypyrrole (PPy), a cost-effective and easily synthesized conductive polymer, holds strong potential for developing biohybrid systems.^20,21^ Additionally, PPy is biocompatible: its pyrrole ring can form covalent bonds with bioactive molecules, which enables diverse functionalities across various applications.^21–23^

To develop a biohybrid system in MFCs, herein we propose modifying electrodes with BP or BP and PPy, where the interactions at the nanomaterial-microorganism interface are novel. To achieve this, we functionalized the surface of a graphite plate anode (GP) with a BP (stabilized with PEDOT:PSS) and PPy nanocomposite by using three strategies: (i) GP electroactivation (EGP), (ii) EGP surface modification with BP (EGP/BP), and (iii) additional EGP/BP modification with polypyrrole (PPy) (EGP/BP/PPy), to obtain MFC1, MFC2, and MFC3, respectively, after a biofilm from mangrove sediment was formed on the electrode upon feeding with sodium acetate.

We aimed to develop biohybrid electrodes that perform good EET and can thus recover higher electron flux from a mixed microorganism culture. We discuss how carbon-surface modifications change EET and the biotic-abiotic interface. For this purpose, we carried out morphological, electrochemical, and microbiological characterizations of all the anodes (EGP, EGP/BP, and EGP/BP/PPy), with and without biofilm.

## Experimental Section

### Chemicals and Solutions

All the chemicals were analytical grade and were used as received. Aqueous solutions were prepared by using ultrapure water from a Milli-Q system (Millipore). Black phosphorus (BP, 100.0%) was obtained from Smart Elements. Potassium ferricyanide (K_3_Fe­(CN)_6_, 99.0%), potassium ferrocyanide (K_4_Fe­(CN)_6_, 99.0%), and nitric acid (HNO_3_, 65.0%) were obtained from Qhemis. Poly­(3,4-ethylenedioxythiophene)-poly­(styrenesulfonate) (PEDOT:PSS, 100.0%), pyrrole (Py, 100%), potassium chloride (KCl, 99.5%), and sulfuric acid (H_2_SO_4_, 95.0–98.0%) were purchased from Sigma-Aldrich. Sodium acetate (SA, 99.0%), sodium hydrogen phosphate dihydrate (Na_2_HPO_4_·2H_2_O, 99.5%), sodium dihydrogen phosphate monohydrate (NaH_2_PO_4_·H_2_O, 99.0%), and ethanol (100.0%) were obtained from Merck.

### Synthesis of BP-PEDOT:PSS Suspension and Electrode Modification Procedures

Briefly, BP with PEDOT:PSS was synthesized by following the procedure described by Souza et al.^24^ For this purpose, 0.05 g of BP crystals were mixed with 0.01 g of PEDOT:PSS in 10.0 mL of ethanol. The mixture was sonicated in an ice–water bath for 6 h, and a uniform suspension of 0.48 mg mL^–1^ BP-PEDOT:PSS was achieved. This suspension was subsequently used to modify EGP.

First, GP (3.3 × 2.8 × 0.5 cm) was polished with alumina; then, it was washed and sonicated with ultrapure water twice. Next, it was immersed in 1.0 mol L^–1^ HNO_3_, ethanol, and ultrapure water and sonicated for 5 min. GP was electroactivated by cycling (10 cycles) in acidic medium (0.5 mol L^–1^ H_2_SO_4_) over a potential range of −0.5 to 1.2 V at a scan rate of 10 mV s^–1^. The resulting EGP was rinsed with ultrapure water.

After electroactivation, a BP-PEDOT:PSS layer was added to EGP by drop-casting (30 drops) onto both sides of the electrode, resulting in a total mass of 1.95 mg cm^–2^ of BP-PEDOT:PSS deposited. Three sequential steps were conducted, and each layer was allowed to dry before the next one was added. The resulting EGP/BP was analyzed by Raman spectroscopy to confirm the structure of the synthesized BP-PEDOT:PSS composite. For this purpose, a high-performance MicroRaman XploRA PLUS microscope (HORIBA France SAS) with a ×50 VIS-LWD-DF objective lens was used.

Finally, PPy was electrodeposited on EGP/BP from a 0.2 mol L^–1^ pyrrole solution (0.1 mol L^–1^ phosphate buffer, pH 7.0) by cyclic voltammetry within a potential window of 0.0–1.6 V at a scan rate (ν) of 10 mV s^–1^ for three cycles, to give EGP/BP/PPy.

EGP, EGP/BP, and EGP/BP/PPy were characterized by Energy Dispersive X-ray Spectroscopy (EDS) and Scanning Electron Microscopy (SEM) by using an E-prism Scanning Electron Microscope (Thermo Fisher Scientific Inc.).

Cyclic voltammetry was performed to evaluate the modifications introduced on the carbon surface. For this purpose, a 1.0 × 10^–3^ mol L^–1^ [Fe­(CN)_6_]^4–^/[Fe­(CN)_6_]^3–^ solution in 0.1 mol L^–1^ KCl was used as electrochemical probe, and a potential window of −0.3 to 1.2 V vs Ag/AgCl_(sat)_, at a scan rate (ν) of 10 mV s^–1^ was employed.

### MFC Assembly and Operation

EGP, EGP/BP, and EGP/BP/PPy were tested in dual-chamber MFCs, which consisted of an open-air cathode and an anodic chamber (50 mL). The cathode (15 cm^2^), which contained 40% (m/m) Pt (HT1400W, ELAT GDL-BASF), was hot-pressed with pretreated Nafion-117.^24^ All the tests were performed in triplicate.

The inoculum came from a mangrove sediment located in the city of Vitória, state of Espírito Santo, Brazil (Latitude: −20°16′45.3′“S; Longitude: −40°18′27.4”’W). A 50 mL aliquot of a 20.0% (m/v) suspension composed of mangrove sediment and Lovley and Phillips medium^21^ was transferred to the anodic compartment. The Lovley and Phillips medium composition (g L^–1^) was as follows:^25^ NaHCO_3_ (2.5), NaHCO_4_ (0.74), NaHPO_4_ 2H_2_O (0.6), NH_4_Cl (1.5), MgCl_2_ (0.1), MgSO_4_ (0.1), yeast extract (0.05), CaCl_2_ 2H_2_O (0.1), KCl (0.1), NaCl (0.1), NaMoO_4_ 2H_2_O (0.001), and MnCl_2_ 4H_2_O (0.005). SA (2.0 g L^–1^) was added as an organic carbon source, and the pH was adjusted to 8.5.

To accelerate biofilm formation and enrich electroactive microorganisms on the anode surface, 0.8 V was initially applied to the system for 10 days.^26^ After, the voltage was increased to 1.0 V for 3 days. This electrostimulation startup helps select microorganisms that enhance extracellular electron transfer (EET) and reduce acclimation time. To measure the current, a 20 Ω resistor was connected between the anode and the source during the experiments.^27^ After being electrostimulated, the MFCs were connected to a 1000-Ω external resistor, and cell voltage was recorded every 15 min by using an Arduino Mega 2560 board connected to a computer and an Excel (2013) program.

### MFC Electrochemical Performance

After the MFC was operated for 450 h, electrochemical measurements were accomplished on an AUTOLAB potentiostat/galvanostat (PGSTAT 128N controlled by the NOVA 2.1 software). The MFCs were disconnected from the external resistance until the cell voltage reached a stable open circuit voltage (OCV). An Ag/AgCl_(sat)_ electrode served as reference. The anode (EGP, EGP/BP, and EGP/BP/PPy) performance was assessed under abiotic conditions (without biofilm) and after a biofilm emerged on the anodic surface.

Cyclic voltammetry (CV) was conducted over a potential window spanning from −0.6 to 1.0 V, at a scan rate (ν) of 1 mV s^–1^. Chronoamperometric measurements were performed under a constant potential of +0.5 V for 1 h. Power density curves and internal resistances (Rint) were determined by linear sweep voltammetry (LSV) at a scan rate of 1 mV s^–1^. Scans were accomplished from the OCV to 0.0 V, as described in eqs 1 and 2 ^24^ respectively.

1 $$P=\frac{iU}{A}$$

2 $$Rint=\frac{U}{i}-Rext$$

where *P* is the power density (W m^–2^), *i* is the current (A), *U* is the cell voltage (V), *A* is the geometric area of the anode (m^2^), and *Rext* is the external resistance (Ω).

Electrochemical impedance spectroscopy (EIS) analyses were performed at the open circuit potential (OCP). The frequency was varied from 100 kHz to 0.01 Hz, with ten frequency points per decade; a root-mean-square sinusoidal disturbance with an amplitude of 10 mV was used.

### Anode Microbial Diversity

Raw genomic DNA was extracted using the ZymoBIOMICS DNA Miniprep Kit (Zymo Research). The full-length 16S rRNA gene (∼1.6 kb) was amplified using primers 27F and 1492R. Amplicons were verified by agarose gel electrophoresis and quantified prior to library preparation using the SQK-LSK114 kit (Oxford Nanopore Technologies). Sequencing was performed on a Flongle Flow Cell (FLO-FLG114) using the MinION platform.

Raw electrical signals were basecalled into FASTQ format using Dorado (v0.5.3, GPU-accelerated). Reads were quality-filtered using NanoFilt (v2.8.0) with a minimum Phred score of Q ≥ 10. Barcode demultiplexing was performed using Dorado demux (v0.5.3), and adapter/primer trimming was conducted using Porechop ABI (v0.5.0). Additional filtering, clustering, and consensus sequence generation were performed using NGSpeciesID (v0.3.0) with Medaka polishing (v0.11.5).

Taxonomic classification was carried out using KMA (v1.5.0) against a curated in-house 16S rRNA database, with representative sequences further validated using BLASTn (v2.16.0). Low-abundance sequences (<0.1%) were excluded to reduce noise and improve taxonomic confidence.

Sequencing depth ranged from 2,775 to 4,107 reads per sample after quality filtering, with an average of approximately 3,409 reads per sample. Read lengths ranged from 13 to 3,710 bp, with an average of ∼1,154 bp, consistent with full-length 16S Nanopore sequencing.

To ensure comparability among samples, microbial community composition was evaluated based on relative abundance profiles derived from normalized count tables. Additionally, alpha diversity indices were calculated at the species level using the scikit-bio package (v0.7.0), providing an independent assessment of community richness and evenness across samples. Functional inference of microbial communities was performed using PICRUSt2 (v2.3.0_b).

### SEM Analysis of the Bioanodes

Scanning electron microscopy (SEM) was performed by using a Carl Zeiss EVO 50 microscope in the high vacuum mode (10^–5^ Torr). Analyses were performed with an electron beam acceleration voltage of 20 kV and a secondary electron detector (SE). The anode with biofilm was immersed in a 2.0% (m/v) glutaraldehyde solution at 4 °C for 6 h and treated with 1.0% (m/v) osmium tetroxide for 2 h. The biofilm was dehydrated with different water/ethanol ratios and dried to the critical point. Finally, the plate was coated with gold by using the sputtering technique (Bal-tec SCD 050, Fürstenstein, Liechtenstein).

## Results and Discussion

### Electrode Surface Characterization and Electrochemical Performance

Figure S1 (Supporting Information) shows the CV profile for pyrrole electropolymerization on EGP/BP. The current increased sharply above +1.0 VvsAg/AgCl_(sat)_because pyrrole monomers oxidized at the anode, and radical cations emerged.^28^ This effect was more pronounced in the first cycle; in the subsequent cycles, the current decreased because the PPy film progressively covered the EGP/BP surface.^29^

To confirm that the GP surface was consecutively modified, we analyzed the modified surfaces by EDS. This analysis revealed the presence of carbon (0.26 keV), oxygen (0.51 keV), phosphorus (2.01 keV), and sulfur (2.31 keV). For EGP/BP/PPy, we also detected a nitrogen peak (0.38 keV), which was associated with the pyrrole molecular structure (Figure S2 - Supporting Information). To confirm that the BP-PEDOT:PSS nanocomposite was formed, we resorted to Raman spectroscopy. We discuss the Raman spectra (Figure S3) in Supporting Information.

Figure 1 presents the SEM images obtained for the anodic surfaces before and after the modifications, as well as in the presence and absence of biofilm.

**Figure 1:**
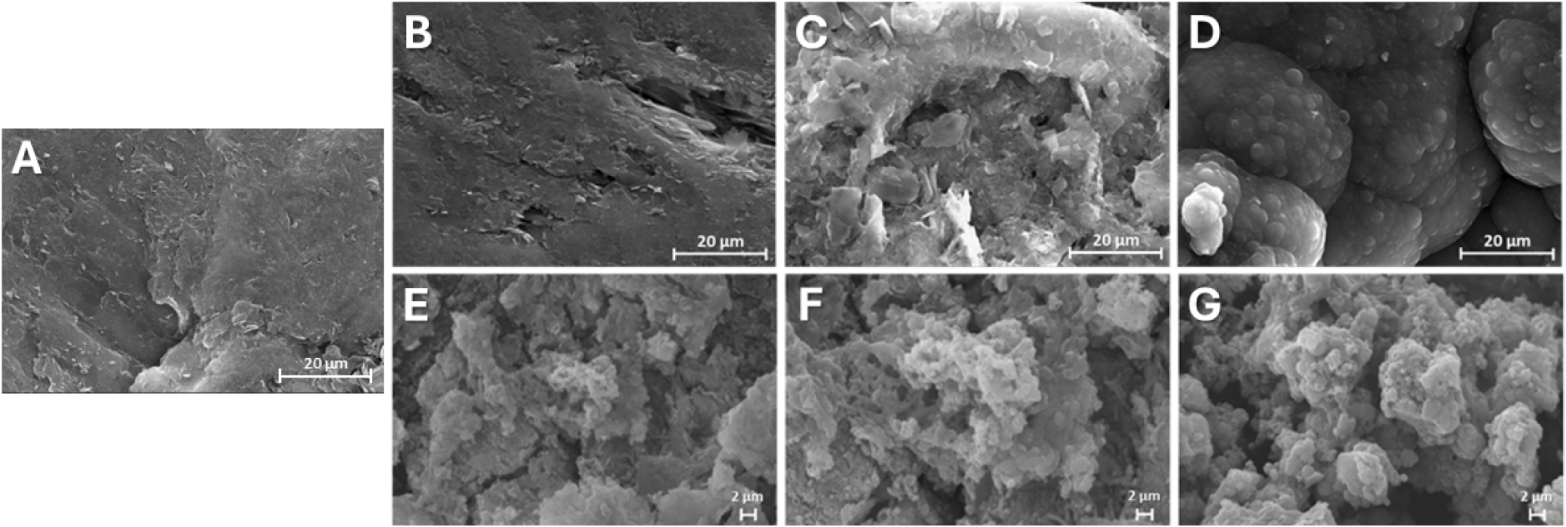
SEM images of the electrode surfaces before and after the modifications, as well as in the absence (A–D) and presence (E–G) of microbial biofilm, respectively: GP (A), EGP (B and E), EGP/BP (C and F), and EGP/BP/PPy (D and G).

Before the biofilm was formed, GP and EGP exhibited smooth and porous morphology (Figure 1A and 1B, respectively). The EDS spectrum of GP revealed a characteristic carbon peak at 0.26 keV. For the EGP, the EDS spectrum of revealed a characteristic carbon peak at 0.26 keV and an additional peak at 0.51 keV, which corresponded to oxygen (Figure S2B - Supporting Information). This resulted from electroactivating GP in acidic medium, which caused oxygenated groups, such as C=O, −C–O/–OH, and COOH/C­(O)­O, to arise on the carbon surface^30^ EGP/BP (Figure 1B) and EGP exhibited distinct morphologies: small and dispersed BP nanoflake structures appeared in the SEM image of the former.^19^ Finally, the SEM image of EGP/BP/PPy (Figure 1C) showed compact globular formations typical of PPy films.^26^

Figure S4 (Supporting Information) presents the EDS elemental mapping of each modified anode. Overall, the elements exhibit a homogeneous distribution across the entire GP surface, indicating uniform coverage and deposition of the modifying materials. Finally, all these results greatly confirm that all modification procedures performed actually modified the surface of the GP electrode.

In the presence of the biofilm, EGP, EGP/BP, and EGP/BP/PPy exhibited similar morphology. The presence of microorganisms coated with an extracellular matrix was evident. This corresponds to the presence of extracellular polymeric substances (EPS) consisted of polysaccharides, proteins, and, in some cases, electrochemically active compounds.^10^ Previous studies have reported that EPS help biofilms to form and to attach to solid surfaces and to protect microbial cells.^10^

In Figure S5 (Supporting Information), we used the [Fe­(CN)_6_]^4–^/[Fe­(CN)_6_]^3–^ redox probe to evaluate how the abiotic anodes performed electrochemically. Although two well-defined redox peaks emerged, the anodic and cathodic peaks of the EGP surface were widely separated (ΔE = 560 mV), which indicated a quasi-reversible electrochemical behavior (Figure S5A - Supporting Information). The anodic (Ia) and cathodic (Ic) peak currents were 8.24 ± 0.39 and −7.52 ± 0.32 mA, respectively.

After BP was deposited on the anodic surface, the voltammogram of EGP/BP exhibited the well-known reversible behavior of the [Fe­(CN)_6_]^4–^/[Fe­(CN)_6_]^3–^ redox probe (Figure S4A - Supporting Information). This behavior reflected enhanced conductivity because BP has intrinsically conductive nature.^31^ The effective surface area also increased, which resulted in higher peak currents (Ia = 12.4 ± 0.48 mA and Ic = −12.6 ± 0.53 mA) and smaller peak-to-peak separation (ΔE = 178 mV). In contrast, PPy film deposition on EGP/BP, to give EGP/BP/PPy, almost suppressed the characteristic redox peaks of the [Fe­(CN)_6_]^4–^/[Fe­(CN)_6_]^3–^ probe and led to wider peak separation (ΔE = 758 mV), which can be attributed to compact polymer layers being formed on the surface that limits the diffusion of the electrochemical probe, leading to attenuated redox peaks. (Figure S5B - Supporting Information).

### Voltage Output Profiles of Modified Anodes in MFCs

Figure 2 compares the voltage profiles of the MFCs bearing an EGP, EGP/BP, or EGP/BP/PPy anode after the biofilm was electrostimulated. We operated the MFCs under 1000-Ω external resistance over five feeding cycles with 2.0 g L^–1^ SA.

**Figure 2:**
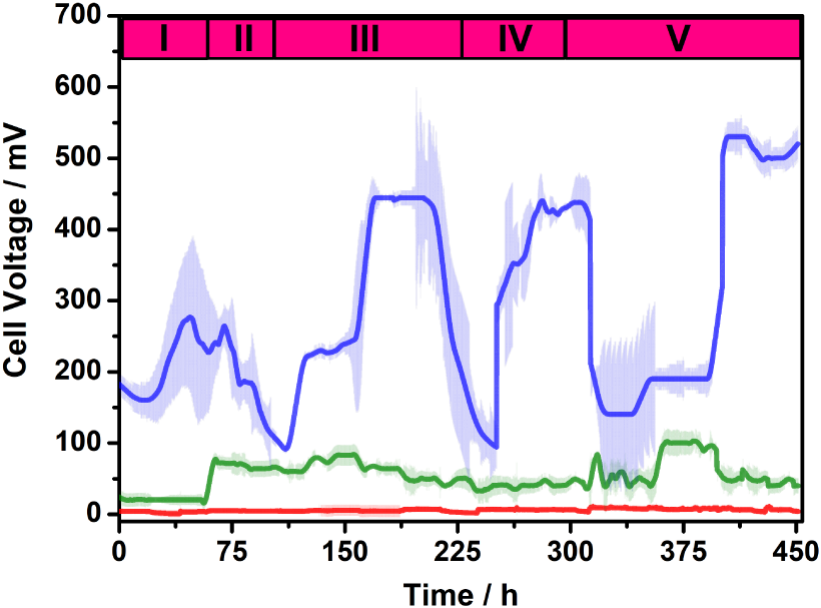
Voltage output during operation of the MFCs fed with SA (2.0 g L^–1^) at each feeding cycle. MFC anode: EGP­(―(red)), EGP/BP (―(green)), and EGP/BP/PPy (―(purple)). Rext = 1000 Ω.

MFC3 had the most modified anode (EGP/BP/PPy) and generated the highest voltage: 276.6 ± 105 mV in the first cycle. While we operated the MFC, the voltage increased gradually, and cycles IV and V reached maximum cell voltages of 439.9 ± 24 and 530.0 ± 35 mV, respectively, and remained stable for prolonged periods.

The absence of PPy in the biohybrid anode led to lower voltage values, poorly defined cycles, and minimal fluctuations. In the case of MFC2 (EGP/BP as anode), the cell output was just 23.9 ± 7 mV during the first 58 h (Cycle I) and oscillated between 24.2 ± 10 and 100.0 ± 18 mV. In contrast, MFC1 (EGP as anode) consistently showed lower voltages (less than 10.8 mV ± 2 mV).

MFC3 had approximately 5.3- and 49.1-fold higher maximum performance than MFC2 and MFC1, respectively. The PPy coating in EGP/BP/PPy in MFC3 allowed more energy to be recovered during acetate-driven EET by serving as a conductive interface between the microbial cells and the anodic surface. Furthermore, the modifications performed on the GP surface provided the anode with higher conductivity and a larger surface area, thereby favoring charge transport and reducing charge-transfer resistance. These effects were further complemented by the pseudocapacitive properties of PPy.^29^ We cannot disregard the fact that PPy has globular morphology and is chemically compatible with microorganisms, which allows microbes to adhere to/to colonize the electrode surface. As we will discuss later herein, we observed that distinct microbiota emerged as we chemically modified the anodic surface.

### Electrochemical Response of Functionalized Electrodes in MFCs

After the MFCs had been operated for 450 h, we constructed polarization and power density curves for them (Figure 3).

**Figure 3:**
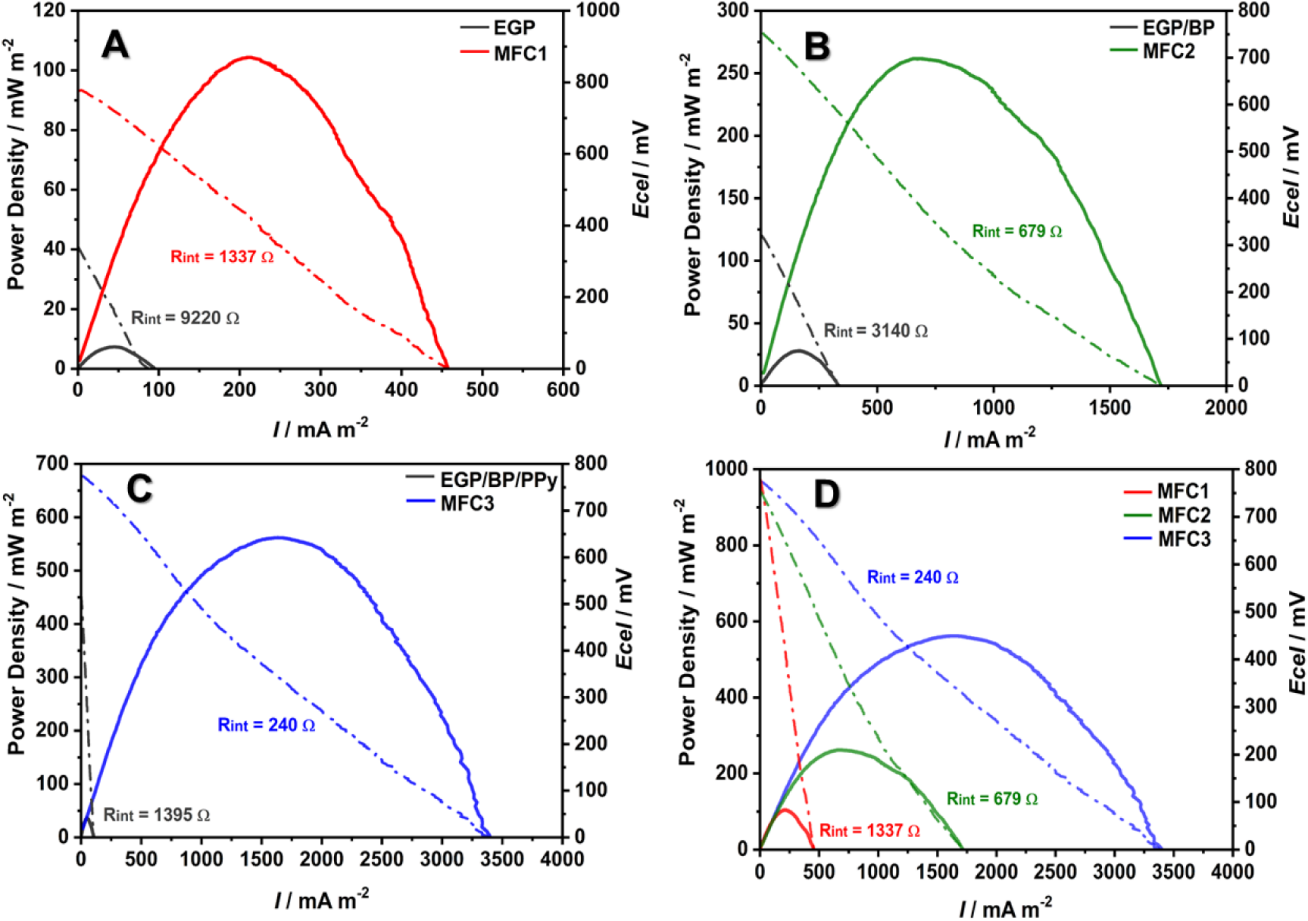
Polarization (**−·–**) and power density curves (—) obtained for the MFCs before and after a biofilm emerged: EGP (A), EGP/BP (B), EGP/BP/PPy (C), and comparison of all MFCs (D). Support electrolyte: Lovley and Phillips medium (pH = 8.5) and [SA] = 2.0 g L^–1^. Scan rate (ν) = 1 mV s^–1^.

Irrespective of the modification introduced on the carbon surface, biofilm formation enhanced EET and contributed to more electrons being collected. This confirmed that an electroactive biofilm arose (Figure 3A–C).

In agreement with the voltage output data (Figure 2), modifying MFC3 provided the highest power density: 561.7 ± 24 mW m^–2^, which corresponded to a 2.2- and 5.4-fold increase compared to MFC2 (261.7 ± 17 mW m^–2^) and MFC1 (104.4 ± 11 mW m^–2^), respectively (Figure 3D). Internal resistance measurements confirmed this result—MFC3 displayed the lowest internal resistance (240 ± 28 Ω), approximately 2.8 and 5.6 times lower than the internal resistance obtained for MFC2 (679 ± 21 Ω) and MFC1 (1337 ± 39 Ω), respectively.

Figure 4 shows the Coulombic charge obtained for each system under both abiotic and biotic conditions and calculated by integrating the respective chronoamperometric curves (Figure S6 - Supporting Information). MFC3 had the highest Coulombic charge (11.68 ± 3.74 mC), which corroborated its improved electrochemical activity. This value was approximately 5.4 and 9.1 times higher than the Coulombic charge obtained for MFC2 (2.16 ± 1.81 mC) and MFC1 (1.28 ± 1.33 mC), respectively.

**Figure 4:**
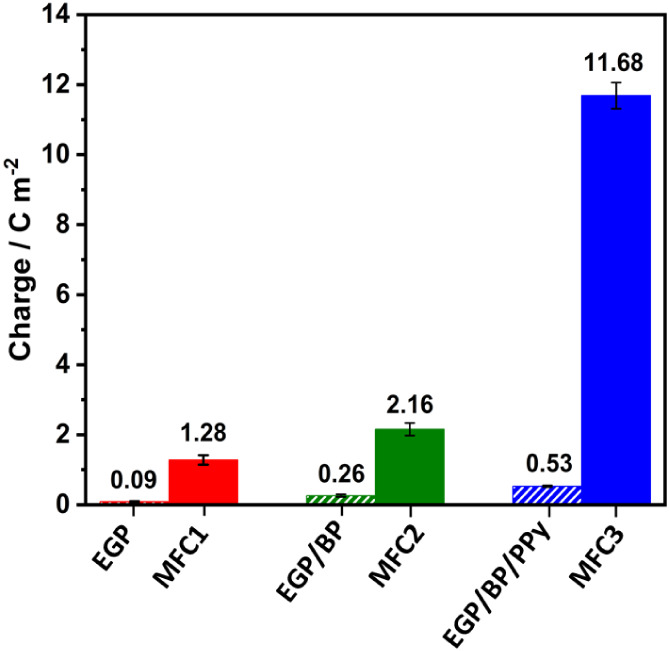
Comparison of Coulombic charges calculated by integrating the respective chronoamperometric curves, before and after the biofilm was formed.

Among the modifications we introduced on the anodic surface, combining BP and PPy contributed to increasing the current the most. In PPy, electrons are delocalized due to the degree of π–π* conjugation.^32^ Besides that, the PPy molecular structure facilitates electron movement along the chain, so that electrons act as charge carriers and provide the electrode with outstanding electrical conductivity.^32,33^

Nevertheless, the physicochemical modifications we applied to the anodic surface increased the surface and electroactive area and provided denser and more stable conductive biofilms. Table 1 summarizes the reported power densities achieved for MFCs containing anodes modified with PPy and its composites.

**Table 1:** Reported Power Densities of MFCs Containing Different PPy-Modified Carbon-Based Anodes^a^

MFC	Substrate	Anode	Modifier	Power density (control) (mW m^–2^)	Power density (modified) (mW m^–2^)	Increased power density^b^	refs
DCMFC	Acetate	CC	PPy-R	15.2	29.3	1.9	^34^
DCMFC	Acetate	GR	PPy	7.0	25.0	3.5	^35^
DCMFC	Synthetic wastewater	GS	MoO_3_/PPy	115.0	380.0	3.3	^36^
DCMFC	Synthetic wastewater	GS	PPy	115.0	230.0	1.7	^36^
BMFC	Marine sediment	CF	PPy/Fe_2_O_3_	69.2	170.4	2.5	^32^
DCMFC	Acetate	GP	EGP/BP	104.4	261.7	2.5	This study
DCMFC	Acetate	GP	EGP/BP/PPy	104.4	561.7	5.4	This study

a DCMFC: dual chamber MFC; BMFC: benthic MFC; CC: carbon cloth; GR: graphite rod; GS: graphite sheet; CF: carbon felt; GP: graphite plate; PPy-R: polypyrrole with globular-like morphology; MoO_3_/PPy: molybdenum oxide/polypyrrole.

b Compared to unmodified anode.

The acetate-fed EGP/BP/PPy yielded up to 5.4-fold enhanced results. In general, PPy-based modifications provide more electrochemically active and biocompatible carbon-based anodes, leading to higher power outputs than unmodified materials. Studies on graphite and carbon cloth anodes^34,36^ have reported that power density improves moderately. These anodes typically provide an increase of between 1.5- and 2.5-fold depending on the conductive support and the PPy composite nature.

Although variations in reactor design, substrate type, and electrode configuration make direct comparisons difficult, the consistent enhancement observed across studies highlights that PPy-based materials are effective anode modifiers in MFC systems.

### Electrochemical Characterization of MFCs

We estimated the anodic and cathodic potentials during one operational cycle (Figure S7 - Supporting Information). The anodic and cathodic potentials remained constant for over 100 h, which reflected that the MFCs were stable. MFC3 had the most negative anodic potential (−350 ± 26 mV vs Ag/AgCl_(sat)_), while MFC2 and MFC3 had similar anodic potentials: −290 ± 15 and −297 ± 11 mV, respectively. The experimental and calculated cell voltage values agreed well, especially at the end of the measurement.

Cyclic voltammograms of the biofilm-covered anodes revealed that the voltametric area increased, particularly for MFC3 (Figure 5), which indicated that the electrochemical activity was enhanced, and that microbes colonized the anodic surface.^37^ The larger cyclic voltammogram area suggested higher capacitive behavior and improved charge transfer at the biofilm-anode interface. This likely resulted from the synergistic BP and PPy effects, which allowed the microorganisms and the conductive substrate to exchange electrons more easily. In the absence of biofilm, all the anodes showed nonelectroactive behavior.

**Figure 5:**
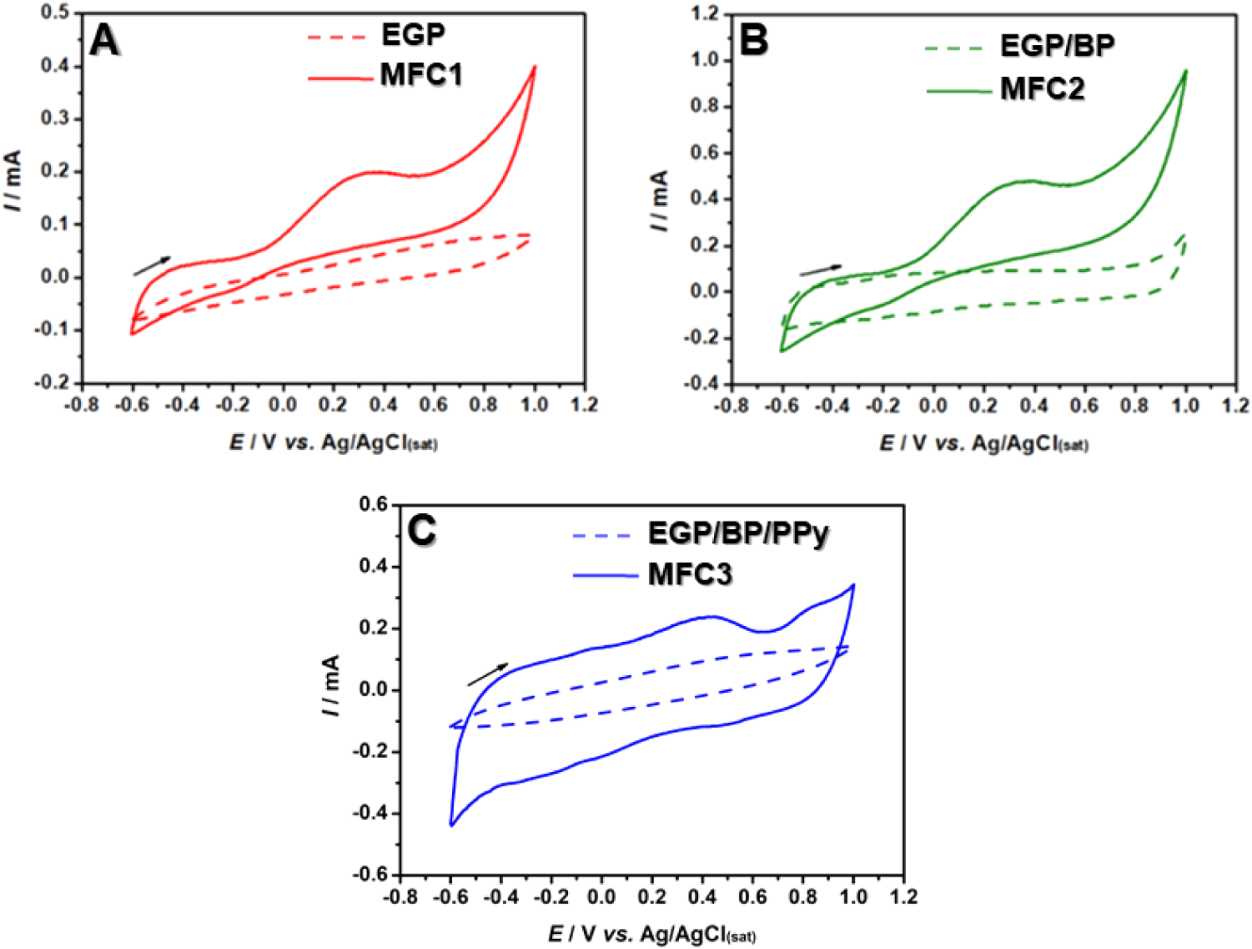
Cyclic voltammograms obtained in the absence (dashed line) and presence (straight line) of microbial biofilm: EGP (A), EGP/BP (B), and EGP/BP/PPy (C). Support electrolyte: Lovley and Phillips medium (pH = 8.5) and [SA] = 2.0 g L^–1^. Scan rate: 1 mV s^–1^.

For MFC1 and MFC2, we identified a well-pronounced anodic peak around 0.25 V vs Ag/AgCl_(sat)_. This peak shifted to 0.40 V in the case of MFC3, with EGP/BP/PPy as anode, indicating changes in the electrochemical processes occurring at the electrode surface, more precisely at the electrode-biofilm interface. The cathodic peak was much less pronounced and appeared at −0.20 V. These results confirmed that an electroactive microbial biofilm emerged on the anodic surface. The biofilm originated from oxidation and reduction of electroactive species (e.g., cytochrome)^38^ in the microorganism cell wall and of species produced by the microorganisms, respectively.^39^

Once again, the modifications performed on the GP surface provided the anode with higher conductivity and a larger surface area, thereby favoring charge transport and reducing charge-transfer resistance. In addition, modifying the GP surface led to a rougher and more porous anode, which in turn may have caused microbial cells to adhere better to the anodic surface.

We conducted EIS analyses at the OCP (Figure 6) to obtain more detail of the interface electrode/biofilm. All EIS complex plane graphs in the presence and absence of the biofilm for all modification introduce showed a semicircle, related to the charge transfer reaction, at high frequencies a straight line, related to the diffusion-limited oxidation process, at low frequencies.^40^ We conducted EIS analyses at the OCP (Figure 6) to obtain more detailed information on the interface electrode/biofilm. The solution resistance (Rs) ranged from 5.56 to 10.0 Ω. All EIS complex plane graphs, in the presence and absence of the biofilm, for all modifications introduced showed a semicircle, related to the charge transfer reaction, at high frequencies; and a straight line, related to the diffusion-limited oxidation process, at low frequencies.^40^ The EIS complex plane graphs obtained were analyzed and fitted to an equivalent electrical circuit compatible with the electrochemical behavior of the system (Figure 6D). In this circuit, Rs represents the ohmic resistance of the electrolyte solution, mainly due to ionic conduction. The CPE (Constant Phase Element) represents the nonideal capacitive behavior of the electrical double layer formed at the anode interface, reflecting surface heterogeneities and the nonuniform distribution of active sites. The parameter Rct refers to the charge-transfer resistance, which is directly related to the kinetics of electrochemical reactions at the electrode/electrolyte interface. Finally, W_0_ corresponds to the finite Warburg impedance element associated with diffusion-limited, confined mass transport at the anode interface, indicating restrictions on the transport of electroactive species in the microbial film.^40,41^

**Figure 6:**
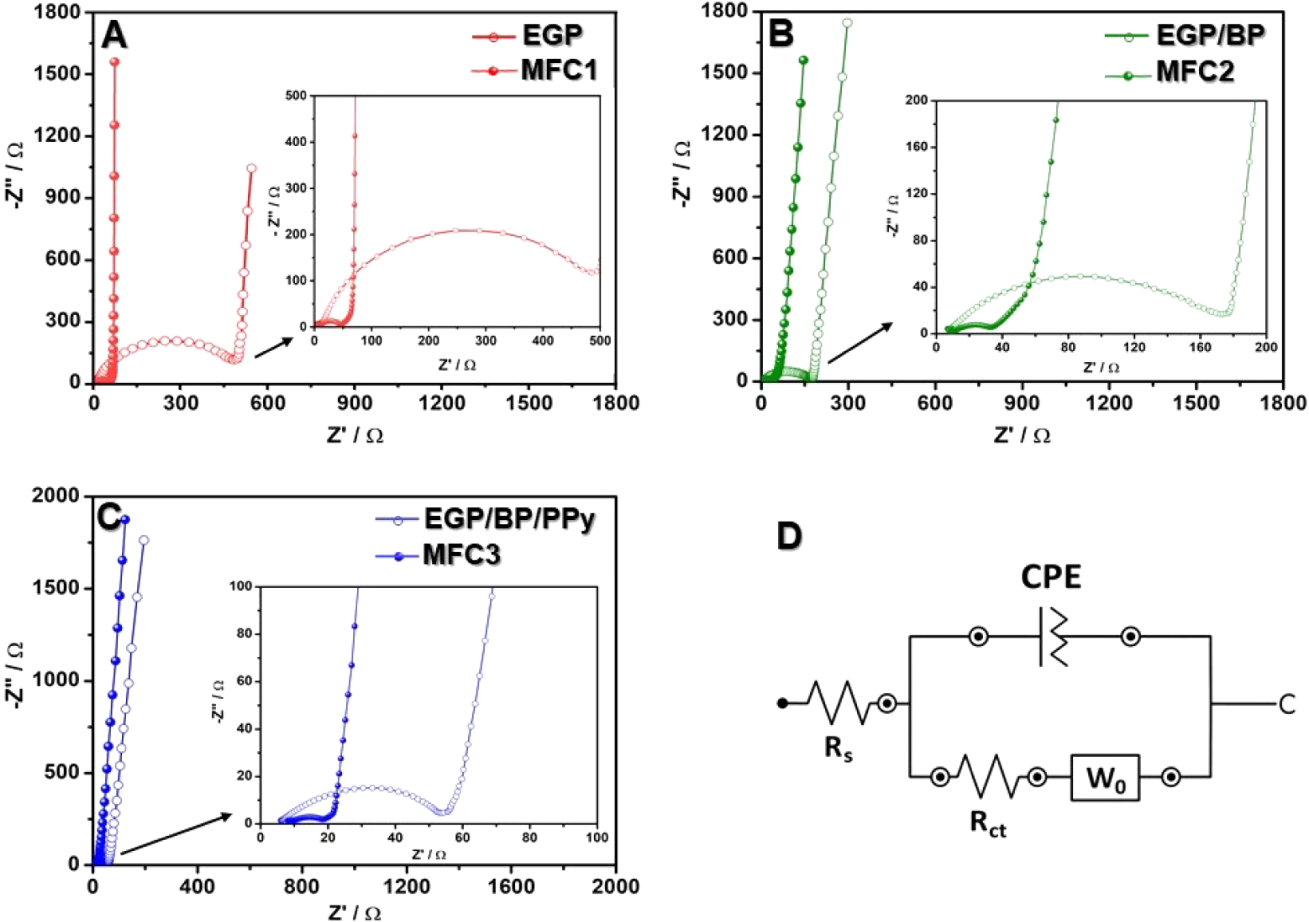
EIS spectra obtained for EGP (A), EGP/BP (B), and EGP/BP/PPy (C) before and after microbial biofilm formation, and (D) equivalent circuit proposed.

In the absence of biofilm, the EIS spectra of the complex showed that the charge transfer resistance (Rct) decreased as we modified the anodic surface, which was accompanied by improved mass transport from the electrolyte to the electrode interface. EIS parameters obtained for the different anodes before and after microbial biofilm growth is shown in Table S1. The average error ranges from 1 to 2%.

EGP exhibited the highest Rct (513.6 ± 65 Ω). Subsequent BP (EGP/BP) and PPy (EGP/BP/PPy) incorporation reduced Rct to 152.6 ± 42 Ω and 46.0 ± 23 Ω, respectively. Therefore, the applied surface modifications effectively reduced Rct, confirming that BP and PPy had higher intrinsic conductivity than EGP. This behavior indicates that the biofilm had good conductivity and effective cooperative EET behavior. We also observed differences at the biofilm interface when the anode is modified. The Rct of the MFCs decreases as follows: MFC3 (14.1 ± 6 Ω) ≪ MFC2 (29.5 ± 10 Ω) ≪ MFC1 (36.8 ± 16 Ω), which could explain why MFC3 presented the highest current density. The lowest charge-transfer resistance in MFC3 confirmed that the synthesized composite enhanced the catalytic efficiency of the bioelectrochemical reaction (Table S1, Supporting Information).

Successive modifications and biofilm formation increased the double-layer capacitance (Cdl) slightly (Table S1 - Supporting Information). On the basis of Cdl, we estimated the electrochemical surface area (ECSA) by using eq 3.^42^

3 $$\mathrm{ECSA}=\frac{\mathrm{Cdl}}{\mathrm{Cs}}$$

where Cdl is the double layer capacitance, and Cs is the specific capacitance of graphite (0.0043 mF cm^–2^).^24^

Given that capacitance is approximately proportional to the area available for charge to accumulate, the ECSA values for anodes containing biofilm were 58.5 ± 0.7 cm^2^ for EGP, 65.6 ± 1.4 cm^2^ for EGP/BP, and 137.6 ± 3.0 cm^2^ for EGP/BP/PPy. In contrast, the corresponding ECSA values for anodes without biofilm were 36.1 ± 1.2 cm^2^, 51.5 ± 1.6 cm^2^, and 749.2 ± 0.9 cm^2^, respectively. This comparison shows that ECSA is altered the for each modification type. Decreased Rct can be attributed to the combined effects of increased ECSA, the high electronic conductivity and rapid charge-transfer characteristics of BP and PPy,^18^ and the arising of an electroactive and conductive microbial biofilm. The EIS results corroborated the other findings of this study and confirmed that modifying the anodic surface increased the surface area and electronic conductivity.

### Biofilm Microbial Communities

We analyzed the microbial community of each MFC on the basis of 16S rRNA gene sequencing and employed canonical correspondence analysis (CCA) to evaluate how modifying the anode impacted the microbial community structure in the MFCs (Figure 7A and 7B, respectively).

**Figure 7:**
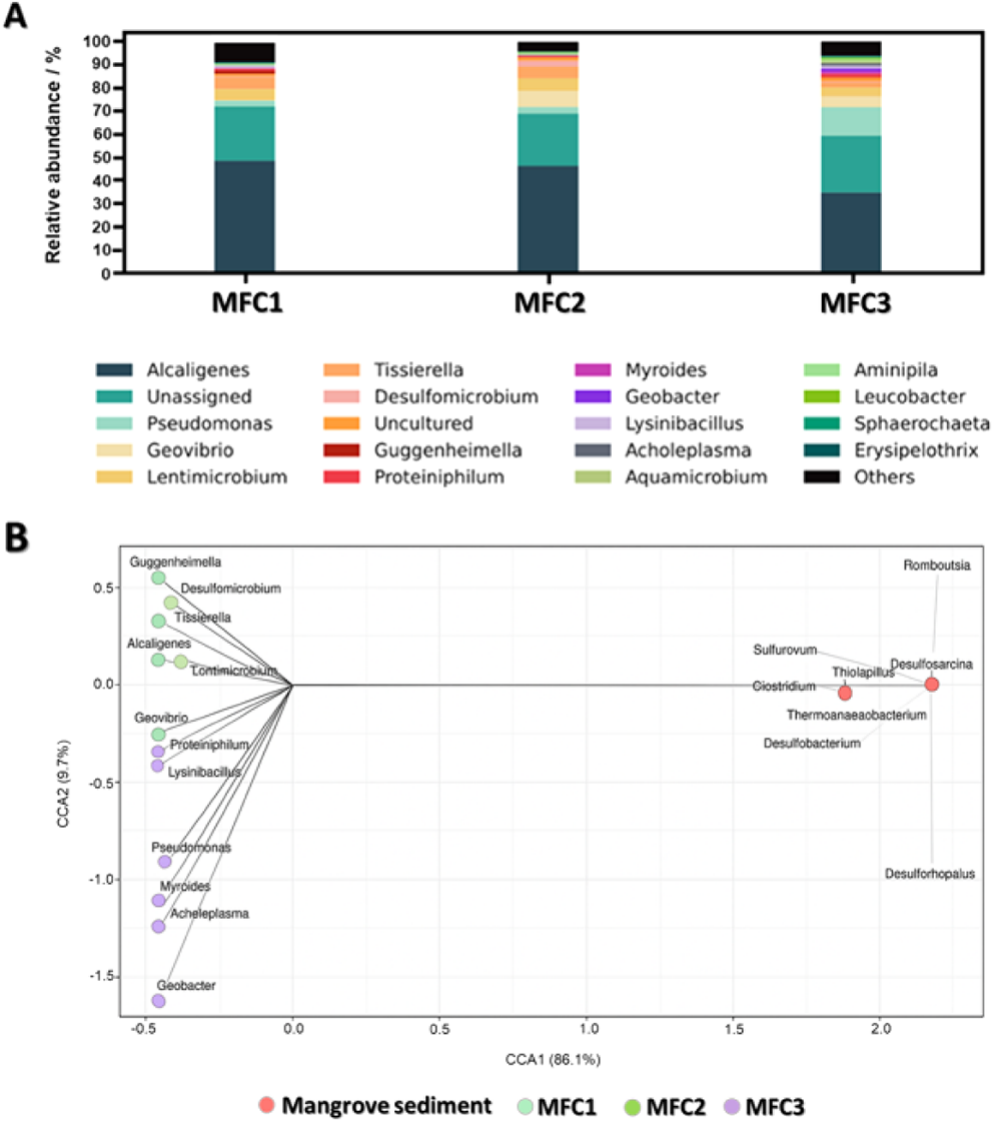
(A) Relative abundance of bacterial genera in MFC_EGP, MFC_EGP/BP, and MFC_EGP/BP/PPy, and (B) canonical correspondence analysis (CCA).

*Alcaligenes* was the most abundant genus in the biofilms and represented 49.1%, 46.7%, and 35.1% of the bacterial communities in EGP, EGP/BP, and EGP/BP/PPy, respectively (Figure 7A). This genus, which belongs to the phylum *Proteobacteria*, has previously been reported in MFC biofilms, where it plays important roles in biodegradation and bidirectional EET mediated by pili and outer membrane proteins.^13,43^

Although the genus *Alcaligenes* remained dominant, the other taxa differed. MFC1 also contained *Tissierella* (5.0%), *Lentimicrobium* (4.9%), and *Pseudomonas* (2.4%), but 23.6% of the sequences remained unassigned. MFC2 contained *Geovibrio* (7.0%), *Lentimicrobium* (5.4%), *Tissierella* (4.9%), *Pseudomonas* (2.8%), and *Desulfomicrobium* (2.7%) as well, but 22.6% of the genera remained unassigned. In MFC3, well-known electroactive genera such as *Pseudomonas* accounted for 12.2% of the total microbial community, which was approximately 5.7- and 4.4-fold higher than in MFC1 and MFC2, respectively.

It has been well documented that the genus *Pseudomonas* accomplishes direct electron transfer and indirect external electron transfer. This occurs mainly through production of secondary metabolites, such as pigments, which transport electrons.^39^ The genera *Alcaligenes* and Pseudomonas, which were predominant in this study, have been reported in the literature to exhibit potentially complementary metabolic capabilities in mixed microbial systems.^44^

CCA highlighted that the microbial community structure changed along the anodic modifications, indicating that the anodic composition determined biofilm assembly (Figure 7B). The first axis (CCA1), which accounted for 86.1% of the total variance, clearly separated the communities formed on the modified anodes from the communities in the mangrove sediment (inoculum). This underscored that the electrochemical environments promoted selective enrichment of the biofilm.

The inoculum was associated with genera such as *Sulfurovum*, *Desulfosarcina*, *Clostridium*, and *Thiobacillus*, which are frequently reported in sulfur-rich anaerobic environments, including mangrove sediments characterized by low oxygen availability and the presence of reduced sulfur compounds.^45^

In contrast, the genera *Alcaligenes*, *Tissierella*, and *Lentimicrobium* were closely associated with MFC1 and MFC2, whereas MFC3 showed a higher relative abundance of *Pseudomonas* and the presence of other electroactive genera such as *Geobacter* and *Myroides*, which are well-documented electrogens. Therefore, the improved performance of MFC3 is more plausibly associated with the increased abundance of *Pseudomonas* and the overall synergistic activity of the microbial community, rather than the dominance of a specific electrogenic taxon.

The separation of microbial profiles on the basis of anodic surface modification not only impacts density but also modulates community composition at the genus level. These findings support the concept of functional biofilm engineering, in which specific electrode materials selectively enrich microbial taxa with desirable bioelectrochemical characteristics.

## Conclusions

To improve energy performance through microorganism-electrode interactions, we have proposed three types of anodic modifications in MFCs. Specifically, functionalizing graphite anodes with a BP/PPy nanocomposite effectively improves MFC performance from a bioelectrochemical standpoint. The sequential modifications began with graphite anode electroactivation, followed by BP incorporation on the electroactivated graphite anode surface, and concluded with PPy deposition. These steps afforded biohybrid electrodes with improved EET, increased electron flux from mixed microbial communities, and distinct biofilm formation. Ultimately, the MFC containing the EGP/BP/PPy anode was the most electroactive.

Surface modification of the anode with BP/PPy improved the electrochemical performance of MFCs, leading to higher power output and lower internal resistance compared with the reference electrodes. The modified anodes also showed morphological and electrochemical features consistent with enhanced electrode–biofilm interactions. Collectively, these results strongly demonstrate that hybrid BP/PPy coatings serve as a promising strategy for tailoring anode interfaces in bioelectrochemical systems. Future directions will focus on designing optimized electrode surfaces, integrating MFCs with wastewater treatment or energy recovery systems, and engineering microbial communities to maximize electron transfer efficiency.

## Supplementary Material



## Notes

Additional results including graphs, cyclic voltammograms, chronoamperograms, EDS spectra, Raman analysis, and a table with EIS data (PDF)
