# Supplementary material for: Improving the Biocompatibility and Energy Performance of a Microbial Bioanode with Black Phosphorus and Polypyrrole Nanocomposites
Source: ACS Omega. 2026 Jul 14;11(29):43096–106. doi: 10.1021/acsomega.5c12114 (PMC13425517; doi:10.1021/acsomega.5c12114)
Supplement: Supplementary file 1 [file ao5c12114_si_001.pdf]

SUPPORTING INFORMATION

IMPROVING THE BIOCOMPATIBILITY AND ENERGY PERFORMANCE OF A MICROBIAL BIOANODE WITH BLACK PHOSPHORUS AND POLYPYRROLE NANOCOMPOSITES

João Carlos de Souza[a,b], Ana Clara Bonizol Zani[a,b], Bruna dos Santos Gomes[a], Gustavo Silveira Toldo[a], Valeria Reginatto[a,b], and Adalgisa Rodrigues de Andrade[a,b*]

[a]University of São Paulo (USP), Faculty of Philosophy, Sciences and Letters at Ribeirão Preto (FFCLRP), Department of Chemistry

Avenida Bandeirantes, 3900, Ribeirão Preto - 14040-900, São Paulo State, Brazil

[b]São Paulo State University (UNESP), Institute of Biosciences, Nacional Institute for Identification, Quantification, Dispersion, Environmental Risks, and Mitigation of Pollution by Emerging Contaminants in Marine and Coastal Environments (INCT-CEMAR)

Praça Infante Dom Henrique, s/nº, São Vicente - 11330-900, São Paulo State, Brazil

[*]Corresponding Author

Address: Avenida Bandeirantes, 3900, Ribeirão Preto - 14040-900, São Paulo State, Brazil

E-mail: ardandra@usp.br

Phone: + 55 16 3315-3725

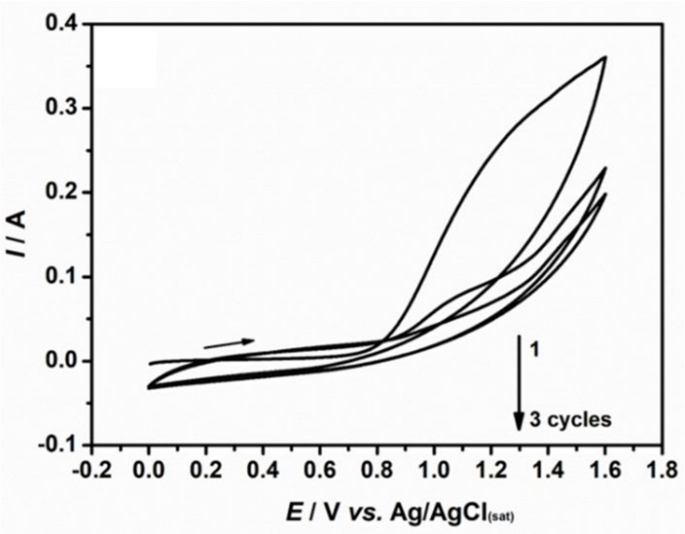

Fig S1. Cyclic voltammograms obtained for electropolymerization of the EGP/BP electrode surface with pyrrole (0.2 mol L^{-1}) in 0.1 mol L^{-1} phosphate buffer at pH 7.0. Scan rate (ν) = 10 mV s^{-1}.

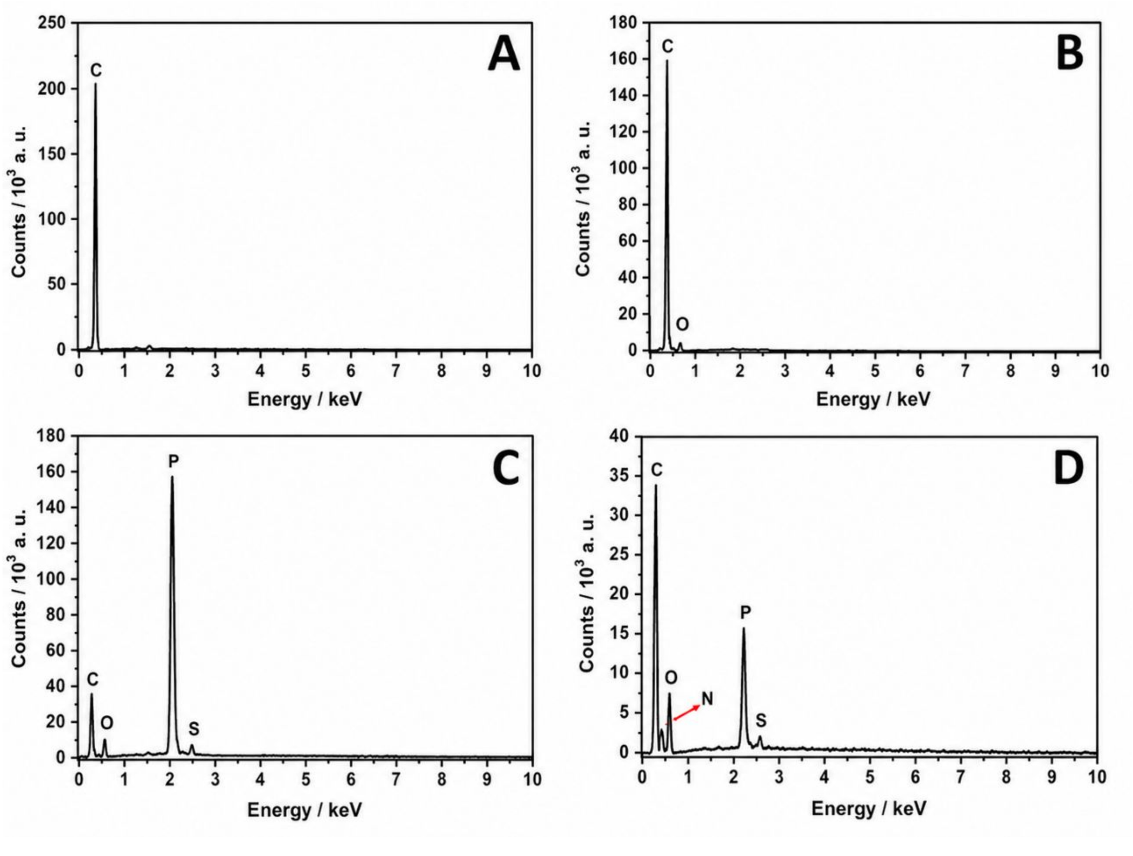

Fig S2. EDS spectra obtained for GP (A), EGP (B), EGP/BP (C), and EGP/BP/PPy (D).

Raman spectroscopy analysis of the composites

The Raman spectrum of BP displayed three characteristic peaks, at 356, 434, and 461 cm^{-1}, which corresponded to the A_g^1, $B^2{}_g$, and A_g^2 vibrational modes of the low-layer BP, respectively.[1] As for PEDOT:PSS, its Raman spectrum showed five main peaks. The peaks at 1528 and 1424 cm^{-1} corresponded to C=C bond asymmetric stretching, while the peak due C=C bond symmetric stretching appeared at 1365 cm^{-1}. The peaks at 1257 and 1232 cm^{-1} referred to stretching of the C-C single bond and the C-C bond between rings, respectively.[2]

Finally, the Raman spectrum of BP-PEDOT:PSS (Fig. S1C) displayed peaks that were very close to the peaks of the BP vibrational modes and the C=C and C-C stretching modes characteristic of PEDOT:PSS. This suggested that the π-π interactions between PEDOT:PSS and BP were not significant,[3] and that BP-PEDOT:PSS was successfully synthesized.

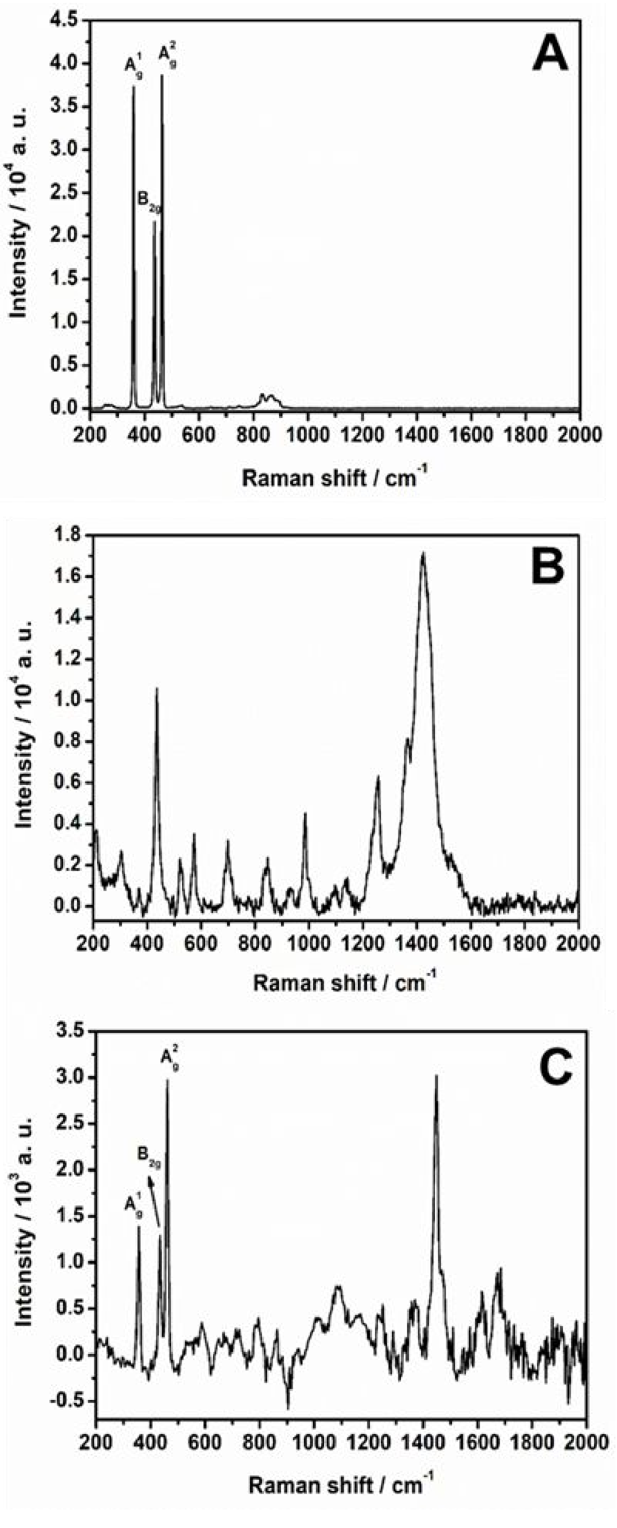

Fig S3. Raman spectra obtained for BP (A), PEDOT:PSS (B), and BP-PEDOT:PSS (C).

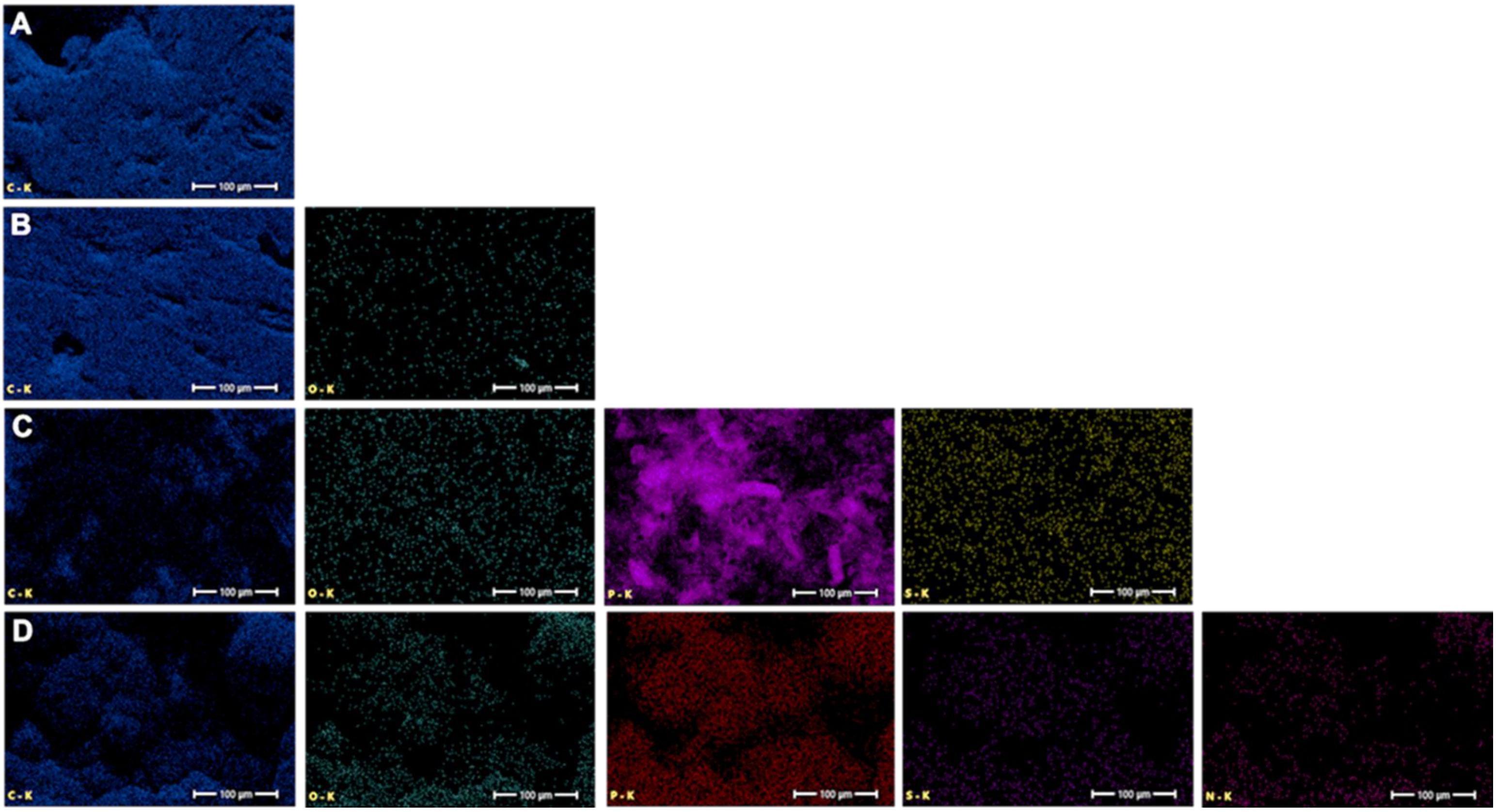

Figure S4. Element intensity images for GP (A), EGP (B), EGP/BP (C), and EGP/BP/PPy (D) electrodes.

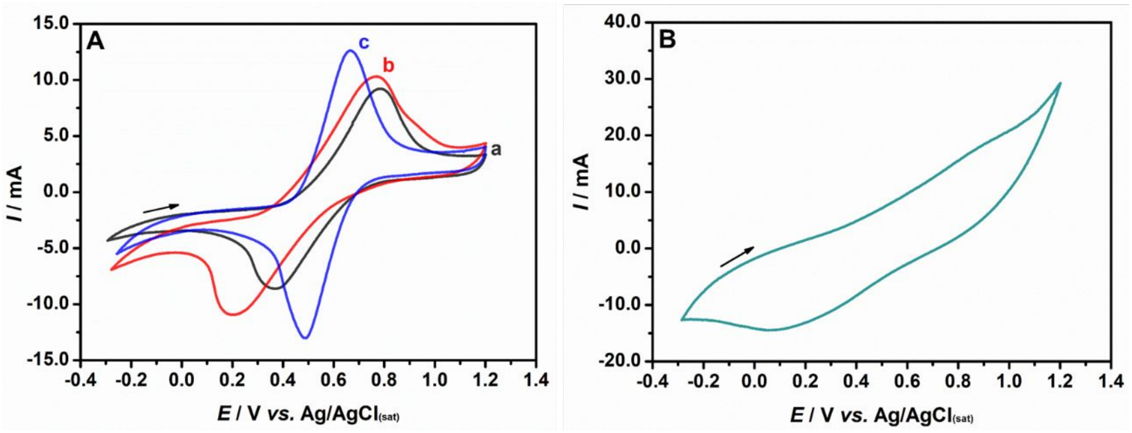

Fig S5. (A) Cyclic voltammograms obtained for GP (black line – a), EGP (red line – b) and EGP/BP (blue line – c) and (B) for EGP/BP/PPy in 1.0×10^{-3} mol L^{-1} $[Fe(CN)_6]^{4-}$/$[Fe(CN)_6]^{3-}$ in 0.1 mol L^{-1} KCl (pH = 7.0). Scan rate (ν) = 50 mV s^{-1}.

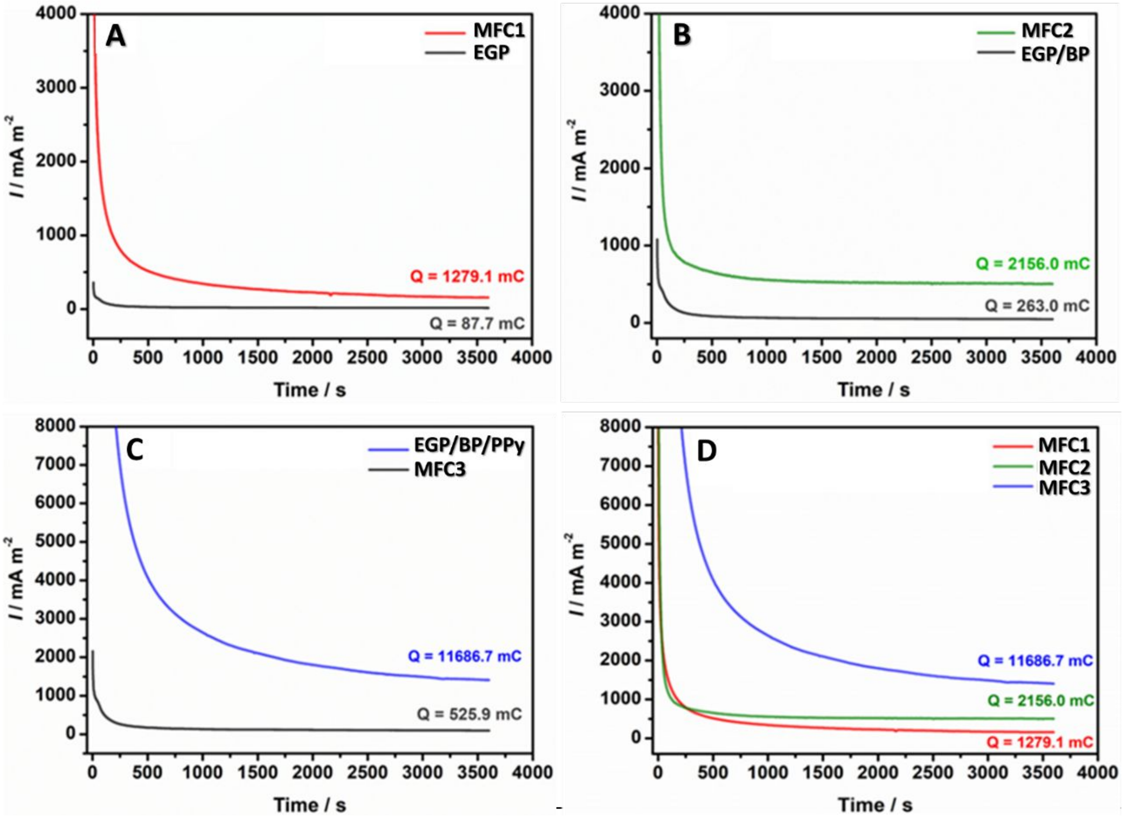

Fig S6. Chronoamperograms obtained for the anodes before and after biofilm formation for the EGP (A), EGP/BP (B), and EGP/BP/PPy (C) in Lovley and Phillips medium and SA (2.0 g L^{-1}). Conditions: potential of 0.5 V *vs.* Ag/$AgCl_{(sat)}$ and duration time of 3600 s.

Anode and cathode profile during cell operation

During one power cycle, we analyzed the experimental potential of the cell at the point of maximum current, which corresponded to the condition of zero external resistance. We compared this value to the theoretical potential difference, which we obtained by directly measuring the anodic and cathodic potentials. We estimated the approximate voltage according to Eq. S1.[4]

$$E_{cell} = E_{cathode} - E_{anode} \quad \textbf{Eq. S1}$$

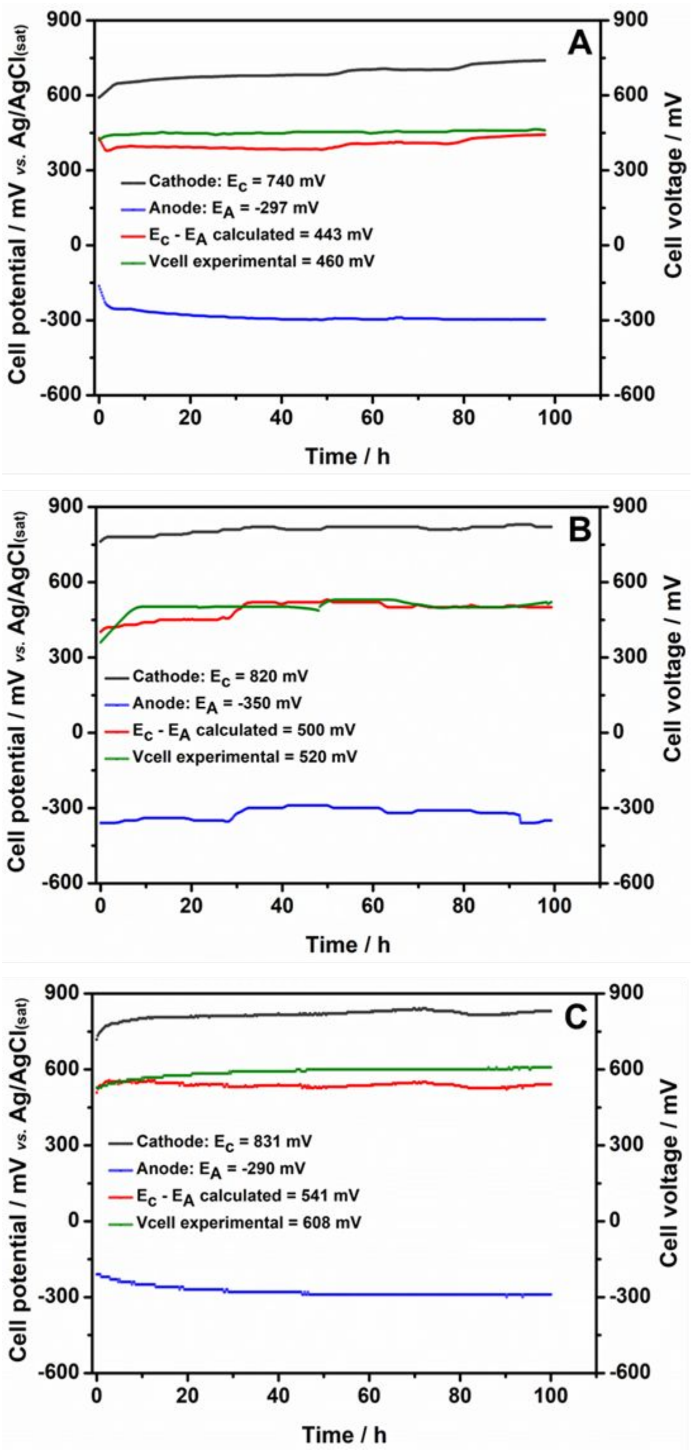

Fig S7. Profiles of the anodic potential (EA), cathodic potential (EC), calculated cell potential (Ecell = EC – EA), and voltage output for MFC1 (A), MFC2 (B), and MFC3 (C).

Table S1. EIS parameters obtained for the different anodes before and after microbial biofilm growth.

Anode	Rs (Ω)	CPE (F)	Rct (Ω)
EGP	10.0	1.55×10^{-4}	513.6
MFC1	7.52	2.52×10^{-4}	36.83
EGP/BP	6.47	2.22×10^{-4}	152.6
MFC2	7.18	2.82×10^{-4}	29.49
EGP/BP/PPy	5.56	5.92×10^{-4}	46.00
MFC3	7.53	3.22×10^{-3}	14.09

References

(1) Mu, H.; Lin, S.; Wang, Z.; Xiao, S.; Li, P.; Chen, Y.; Zhang, H.; Bao, H.; Lau, S. P.; Pan, C.; Fan, D.; Bao, Q. Black phosphorus–polymer composites for pulsed lasers. *Adv. Opt. Mater.* **2015**, *3* (10), 1447-1453. https://doi.org/10.1002/adom.201500336.

(2) Zhang, J.; Ding, W.; Zhang, Z.; Xu, J.; Wen, Y. Preparation of black phosphorus - PEDOT:PSS hybrid semiconductor composites with good film-forming properties and environmental stability in water containing oxygen. *RSC Adv.* **2016**, *6*, 76174-76182, https://doi.org/10.1039/C6RA14762C.

(3) Souza, J. C.; Silva, J. P.; Zanoni, M. V. B.; Andrade, A. R. High sensitive phosphorene and molecular imprinted polymers electrochemical sensor to determine benzene in oilfield-produced water. *J. Environ. Chem. Eng.* **2024**, *12*, 111703. https://doi.org/10.1016/j.jece.2023.111703.

(4) Almeida, É. J. R.; Halfeld, G. G.; Reginatto, V.; Andrade, A. R. Simultaneous energy generation, decolorization, and detoxification of the azo dye Procion Red MX-5B in a microbial fuel cell. *J. Environ. Chem. Eng.* **2021**, *9* (5), 106221. https://doi.org/10.1016/j.jece.2021.106221.
